# Ecological energetics of an imperilled fish: Physiological metrics, experimental protocols and implications for population recovery

**DOI:** 10.1111/jfb.70610

**Published:** 2026-09-06

**Authors:** Jacob W. Brownscombe, Paul A. Bzonek, Kurtis Smith, Lauren E. Rowsey, D. Andrew R. Drake

**Affiliations:** ^1^ Great Lakes Laboratory for Fisheries and Aquatic Sciences Fisheries and Oceans Canada Burlington Ontario Canada; ^2^ Department of Integrative Biology University of Guelph Guelph Ontario Canada

**Keywords:** aerobic scope, bioenergetics, climate change, metabolism, respirometry, species at risk

## Abstract

Insights about fish physiology can be useful for population management, but collecting ecologically relevant data can be challenging, especially for imperilled and sensitive species. With the goal of identifying ecologically relevant physiological metrics, we conducted a range of experiments to characterize the metabolism, food consumption, growth, temperature tolerance and bioenergetics of Silver Shiner (*Notropis photogenis*) from an imperilled population in Canada. We also assessed the potential for collecting some of these metrics with non‐lethal, field‐based studies. Short‐term (≤5‐h) field‐based respirometry generated estimates of standard metabolic rate (SMR) that were statistically similar to 48‐h lab‐based trials, though the abbreviated protocol may still differ from true SMR. A chase‐to‐exhaustion, single‐measurement field respirometry protocol underestimated maximum metabolic rate (MMR) at warmer temperatures relative to longer (24–48 h) laboratory respirometry, possibly reflecting heightened stressor sensitivity at warm temperatures and the limitations of single‐measurement protocols for capturing peak metabolic rate in this small‐bodied species. Silver Shiner exhibited a eurythermal metabolism, with a broad thermal range of high aerobic scope (AS; aerobic capacity above SMR). However, bioenergetics measures including lab growth (observed) and field growth (modelled) showed greater growth potential from 12 to 21°C due to limitations in food consumption capacity >21°C. Survival was also lower above the optimal growth range, supporting links between energetics and stressor sensitivity. Across the metabolic metrics evaluated, a novel metric generated here, aerobic differential (AD = AS − SMR), which subtracts maintenance costs from aerobic capacity, was more closely related to lab and field growth than aerobic scope or factorial aerobic scope. Agitation temperatures from CT_max_ trials also showed some consistency with the onset of energy loss and stressor sensitivity. These findings provide insights into ecologically relevant physiological metrics and experimental designs, and have implications for managing critical habitat. In particular, aerobic differential and agitation temperature show promise as ecologically relevant indicators and have potential as non‐lethal field‐based assessment methods.

## 1 INTRODUCTION

Wild animal populations respond to the ecological conditions of their environment in complex ways, related to how their physiology and behaviour interact with biotic and abiotic conditions (Begon et al., 1990). Characterizing patterns between population dynamics and environmental conditions provides clues to the nature of these complex interactions; however, experimental studies are often necessary to describe the underlying mechanisms (Blumstein & Fernández‐Juricic, 2004; Cooke et al., 2014). To this end, physiological studies under controlled conditions are often insightful and are growing in popularity in both fundamental and applied contexts (Wikelski & Cooke, 2006). Animal physiology is complex, and numerous study approaches are available to inform species management and population recovery (Madliger et al., 2022). Of these, bioenergetics studies are particularly valuable because energy is a fundamental currency of life that can describe patterns of growth and productivity from individuals to populations and entire ecosystems (Brown et al., 2004; Kleiber, 1961). Bioenergetics studies are especially popular and well developed for fishes, with utility demonstrated in diverse applications including habitat management, fisheries management and aquaculture (Brownscombe, Lawrence, et al., 2022; Hansen et al., 1993; Jobling, 1995).

Within the broader field of bioenergetics, metabolism is a key process by which animals mobilize energy. The study of metabolism is a well‐developed field that can describe ecological processes at a range of biological scales (Brown et al., 2004; Kleiber, 1961). For individuals, metabolic rate defines their energetic needs, while their capacity above baseline needs (aerobic scope; AS), calculated from maximum metabolic rate (MMR; maximum metabolic capacity) minus standard metabolic rate (SMR; rate of maintenance metabolism), determines their capacity to conduct essential life processes including movement (e.g., to find food, avoid predators, move to spawning sites) and digestion (Bishop, 1999; Pörtner et al., 2017). AS has been proposed as a key focal metric for assessing fish thermal performance (Pörtner, 2010; Pörtner et al., 2017), yet support for this theory is equivocal as in many cases AS fails to explain the growth optimums and temperature selection of fish. Some have suggested there are more relevant physiological performance measures (e.g., digestive capacity) and derivatives proposed, such as factorial aerobic scope (FAS = MMR/SMR), that can better explain constraints on thermal performance; however, there is limited evidence to date that FAS is an ecologically relevant conversion (Clark et al., 2013; Halsey et al., 2018; Jutfelt et al., 2018). Framed in bioenergetics, AS represents the aerobic capacity available above maintenance; a potential proxy for the energy that can be allocated to activities including foraging, digestion, growth and reproduction (Brownscombe, Raby, et al., 2022). However, AS itself does not directly account for the magnitude of maintenance costs (SMR), which scale with temperature and define the energetic baseline an individual must exceed to achieve net energy gain. Subtracting SMR from AS (or from MMR a second time) yields a metric termed here aerobic differential (AD = AS − SMR, or MMR − 2·SMR) that approximates the surplus aerobic capacity remaining after incurring maintenance costs again as a separate energetic burden, independent from its aerobic capacity calculation. Conceptually, AD penalizes high‐SMR conditions (e.g., warm temperatures) where a larger fraction of any energy intake is committed to baseline metabolism, leaving less for net energy gain. Ultimately, the capacity for individuals to achieve net energetic gain is a key process that is highly relevant to individual‐level biological fitness and population‐level productivity. Thus, there is a need to gain a better understanding of how metabolic metrics like AS, and its derivatives relate to growth rates in the broader bioenergetic context. Further, acute thermal tolerance metrics, critical thermal maximum (CT_max_) and agitation temperature (T_agitation_; Dodsworth et al., 2025) are widely used to characterize upper thermal limits and may inform the temperature ranges over which metabolic and growth metrics remain ecologically meaningful.

Understanding the environmental drivers of population dynamics is of particular importance for imperilled species due to the urgent need for effective conservation actions, including habitat protection (Madliger et al., 2022). Yet, studying these species poses many challenges due to their rarity and the requirement for non‐lethal sampling in many populations. Thus, there is a major need to identify key performance metrics, and experimental approaches to acquire them, that are feasible under non‐lethal sampling constraints. To these ends, we conducted a range of physiological experiments on Silver Shiner (*Notropis photogenis*; Cope, 1865) from a population at the northern edge of its geographic range, where it is listed as a Threatened species (COSEWIC, 2011; Fisheries and Oceans Canada, 2022; Young & Koops, 2013), including field and lab respirometry, acute thermal tolerance (critical thermal maximum; CT_max_) tests and growth assessments. This species was an ideal model for this work because of our proximity to a relatively large, stable wild population within their imperilled range in Canada, enabling a range of experiments to be conducted in the field and lab. Our objectives were to: (1) identify key focal physiological metrics, their inter‐relatedness and ecological relevance, (2) assess the potential for non‐lethal, field‐based studies to generate key physiological metrics and (3) generate insights for the conservation of imperilled Silver Shiner.

## 2 METHODS

### 2.1 Ethics statement

All collection, transport, holding and experimental procedures were conducted per the Canadian Council on Animal Care (AUP # OPA‐ACC‐2022/2023‐15) and *Species at Risk Act* Permit conditions (# 22‐PCAA‐00012).

### 2.2 Data collection

Several experiments were conducted on Silver Shiner, including field‐ and lab‐based respirometry with varied protocols, thermal tolerance studies, as well as food consumption and growth studies across varied experimental configurations to estimate key bioenergetics parameters for this species (Figure 1).

**FIGURE 1:**
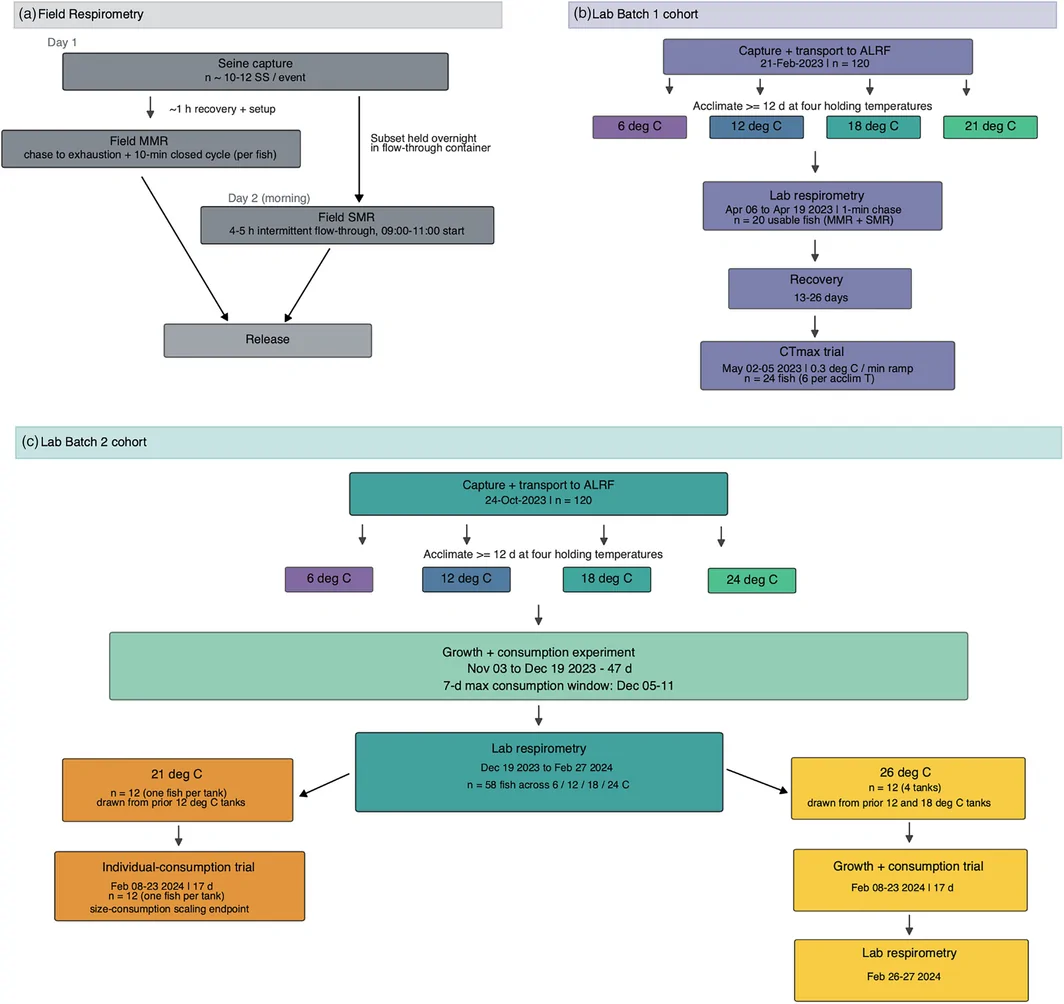
Schematic overview of the experimental designs used in this study to estimate Silver Shiner physiological parameters.

#### 2.2.1 Field‐based metabolism experiments

To assess the potential to measure Silver Shiner metabolism with non‐lethal, field‐based methods, trials were conducted with a mobile respirometer set‐up at a field site beside Sixteen Mile Creek (43.499°N, −79.777°E), Ontario, Canada. Field metabolic rate trials were conducted opportunistically amongst all seasons to collect data across a broad ambient temperature range from 28 August 2021 to 24 August 2023 (3–21°C). Additional sampling, experimental set‐up and protocol details are provided in Appendix S1.

In brief, fish metabolism data were collected via intermittent flow‐through respirometry following standardized techniques (Killen, 2021; Svendsen et al., 2016) over a 2‐day period using a combination of Firesting (Pyroscience, Germany) and Loligo (Loligo Systems Denmark) systems. On the day of capture, field MMR trials were conducted beginning ~1 h after capture and respirometry set‐up, between 1030 and 1430 h. Fish were individually subjected to a commonly used chase‐to‐exhaustion protocol (Norin & Clark, 2016) and placed immediately into size‐matched respirometers for a single 10‐min measurement of metabolic rate. After field MMR trials were completed, and a brief observation period for recovery, the fish were released. For each of the 11 experimental/sampling periods, a subset of captured Silver Shiner (*n* = 10–12) not used in MMR trials were held overnight in the stream in a flow‐through container to allow metabolic rates to decline and any food to be digested, prior to field SMR trials beginning the following day. Fish were transferred directly into respirometer chambers (*n* = 6–8 per day) the morning after capture (0900–1100 h) where they remained for 4–5 h while metabolic rates were recorded and used to estimate SMR. During each field SMR trial, closed/flush cycles varied depending on water temperature to ensure >80% minimum O_2_ saturation during closed cycles. This protocol duration is typically considered too short for true SMR measurement and may be more accurately described as resting metabolic rate (Chabot, Steffensen, & Farrell, 2016); however, longer trials were not logistically feasible at our field site, and we compared our field SMR estimates to longer duration laboratory trials as described below. Upon completion of trials, all individuals were measured for mass and total length and then returned to the capture site. On each SMR measurement day, habitat data at the capture site were also recorded (see Appendix S1).

#### 2.2.2 Laboratory experiments

To conduct metabolism, consumption, growth and CT_max_ laboratory trials, Silver Shiner (*n* = 120 total; *n* = 30 per holding temperature, distributed across multiple experiments described below) were collected from Sixteen Mile Creek on two occasions: February (21 February 2023) and October (24 October 2023), and fish were transported to the Aquatic Life Research Facility (ALRF) at the Canada Centre for Inland Waters (CCIW), Burlington, Ontario. They were initially held at holding temperatures of 6, 12, 18 and 21°C and, throughout all pre‐experimental holding protocols, were fed a diet of bloodworms and brine shrimp to satiation once or twice per day, depending on staff availability. For all fish collection, holding and experimental protocols, extensive methodological details are provided in Appendix S1. Laboratory respirometry trials followed similar methods to the field respirometry trials using the Loligo respirometry equipment; however, protocols were adjusted to extend to 24–48 h at acclimation temperatures from 6 to 26°C (see Appendix S1; Table S1).

To investigate Silver Shiner acute thermal tolerance, a subset (*n* = 24) of the first batch of fish introduced to the lab were subjected to CT_max_ experiments after 10 weeks of holding at experimental temperatures following established best practices (Raby et al., 2025). Fish destined for CT_max_ trials were fed on the same pre‐trial holding schedule and fasted for 24 h before each trial. Trials were conducted on six fish at a time, with fish divided into two 56 L aquariums. Trials were conducted at acclimation temperatures of 6, 12, 18 and 20°C, to span a range of thermal conditions Silver Shiner commonly experience in the wild (Appendix S1; Figure S5). During the trial, water temperature was increased at a target rate of 0.3°C min^−1^ (Desforges et al., 2023). The times and water temperatures for CT_max_ (temperature at loss of equilibrium) and T_agitation_ (temperature at agitation; Dodsworth et al., 2025) were recorded for each fish. T_agitation_ was defined as the temperature at which fish exhibited highly erratic behaviour, agitated swimming and rapid ventilation for >5 s (McDonnell & Chapman, 2015; Raby et al., 2025; Turko et al., 2020). Across all trials, we maintained a warming rate of 0.29 ± 0.1°C min^−1^ (mean ± SD), and a slope *R* ^2^ of 0.99 ± 0.01.

A growth and consumption experiment was conducted with the second batch of fish introduced in the lab, with replicates of *n* = 15 fish per treatment distributed in four tanks per acclimation temperature amongst 6, 12, 18 and 24°C. Further details on the methodology are reported in Appendix S1. After 12 days of temperature acclimation, fish weights were recorded and fish were transitioned to an excess feed diet, fed twice per day on weekdays and once on weekends for a ~7 week (47 days) period. The amount of food provided to each tank was measured each day, and each tank was monitored to ensure excess food was provided (i.e., some food remained un‐ingested after feeding until it was manually removed). In addition, for a subset of days (*n* = 7 consecutively) within this period, the percentage of feed consumed was measured for each tank by collecting and weighing remaining food after 45 min. Upon experiment completion, fish were fasted for 48 h and final mass was measured. This experiment was subsequently repeated at 26°C for 16 days, which was terminated after a shorter duration due to concerns about rapid losses in fish weight. An individual‐level feeding experiment was also conducted concurrently by separating fish into individual tanks (*n* = 12) at 21°C to assess the effect of fish size on consumption capacity for bioenergetics models reported below. The same feeding protocols were followed to estimate food consumption rates. Upon completion of the 21 and 26°C experiments, all fish were fasted for 48 h, euthanized with 250 mg L^−1^ tricaine methanesulfonate and a final mass was measured.

### 2.3 Data analysis

All data analyses were conducted in R (version 4.2.1; R Core Team, 2019), with data manipulation completed in tidyverse (Wickham et al., 2023) and plotting in ggplot2 (Wickham, 2016). All statistical outputs were interpreted as *p* < 0.01 as strong evidence, *p* < 0.05 as moderate evidence, *p* < 0.10 as weak evidence and *p* > 0.10 as no evidence for significant differences (Muff et al., 2022). In all model applications, full models were parameterized with two‐way interactions, and final model structure was determined using backward selection based on Akaike information criterion (AIC) scores. Model fit was assessed in all cases using protocols outlined in Zuur et al. (2009).

#### 2.3.1 Metabolism & CT_max_

Extensive details on respirometry data processing, including quality‐filtered metabolic rate trajectories per individual fish for field (Figure S3) and laboratory (Figure S4) respirometry, are reported in Appendix S1. Fish metabolic rates (MO_2_; mg O_2_ kg^−1^ h^−1^) were estimated from the slopes of oxygen decline within the respirometer chambers, which were corrected for chamber volume and background respiration rates. MMR was considered the highest value observed in each trial. SMR was calculated from lab data by removing the five lowest values, then calculating the mean of the lowest 10% of values (Chabot, Steffensen, & Farrell, 2016). Field SMR data were more limited in sample size, and no obvious low outliers existed, so the mean of the three lowest values was taken as an estimate of SMR.

There were clear differences in the relationships between temperature and MMR between field and lab trials, so lab and field MMR were modelled separately. With field‐based MMR data, linear models were fit to assess relationships between chase time, fish mass, temperature and MMR. Chase time was fit with temperature (third‐order polynomial) and fish mass as predictors. By default, metabolism and fish mass were ln‐transformed prior to modelling to conform to known mass‐scaling relationships (Clarke & Johnston, 1999). MMR was ln‐transformed and fit with chase time, temperature (second order polynomial) and ln‐mass. Lab MMR data were fit with temperature and ln‐fish mass predictors. ln‐SMR was fit with temperature, ln‐mass and data type (field, lab) as predictors. A second linear model was also fit with metabolic rate data converted to g‐O_2_ g‐fish^−1^ day^−1^ to generate estimates at the appropriate scale for bioenergetic models (described below). Some lab trials were limited to 24 h instead of 48 h; this potential effect was assessed by filtering the lab data to include temperatures that contained both durations (16–24°C) and fitting a linear model to MO_2_ with measure duration as a categorical predictor.

Fitted models (lab‐only data for MMR, combined field and lab data for SMR) were used to predict SMR and MMR amongst a range of fish sizes (1–10 g) and water temperatures (1–33°C). With these predicted values, AS was calculated as MMR‐SMR, FAS as MMR/SMR and AD as AS–SMR. CT_max_ trial data were analysed with linear models, with T_agitation_ and CT_max_ as response variables and acclimation temperature and fish mass as predictors. Response ratios for T_agitation_ and CT_max_ were calculated as the rate of change in these metrics per degree of change in acclimation temperature from the lowest (6°C) to highest (20°C) experimental temperatures.

#### 2.3.2 Growth & food consumption

Growth and food consumption data were analysed with a focus on quantifying relevant metrics for bioenergetics modelling below. Fish growth (final mass—initial mass) was fit with a linear model with initial fish mass and water temperature as predictors. All consumption data were expressed in g‐food · g‐fish−1 to match the input convention of Fish Bioenergetics 4.0 (Deslauriers et al., 2017); subsequent bioenergetic outputs were converted to energy units (Joules) using the food energy density parameter (EDP) in Table 1. Fish weights were estimated daily by linear interpolation from measured start and end weights. Maximum consumption rates were derived as the mean consumption value during the latter 4 days of measured feeding rates, described above. Group (tank)‐level consumption data amongst temperatures (6–26°C) were fit with a generalized additive model to identify optimal consumption temperature (CTO; Table 1), as the temperature of the highest fitted consumption value. The consumption coefficient (CQ; rate of increase in consumption with temperature) was calculated as the Q_10_ value between the lowest (6°C) and highest measured (24°C) consumption values, by: CQ = (R2/R1)^10/(T2−T1)^, where R1, R2 were consumption rates, and T1, T2 were corresponding temperatures.

**TABLE 1:** Bioenergetics model parameters.

Parameter	Value	Definition	Source
Oxycal	13,560	Oxycalorific coefficient, conversion from g O_2_ to Joules	Fish Bioenergetics 4.0 (Deslauriers et al., 2017)
ED	5522	Fish energy density (Joules)	*Phoxinus* spp.; (He, 1986; Trudel & Boisclair, 1993)
EDP	2460	Food energy density (Joules), bloodworms in lab	(Meyer et al., 2016)
CA	0.305	Consumption size scaling intercept	Individual consumption experiment
CB	−0.310	Consumption size scaling slope	*Phoxinus* spp.; (He, 1986; Trudel & Boisclair, 1993)
CQ	2.436	Consumption temperature slope	Consumption Q10 (6–24°C), consumption experiment
CTO	23	Consumption optimal temperature (°C)	Maximum consumption temperature experiment
CTM	33	Consumption maximum temperature (°C)	CTmax experiment (maximum CTmax value)
RA	0.00137	Metabolism‐fish mass intercept	Standard metabolic rate, metabolism experiment
RB	−0.277	Metabolism‐fish mass slope	Standard metabolic rate, metabolism experiment
RQ	0.080	Metabolism temperature scalar	Standard metabolic rate, metabolism experiment
ACT	0	Activity costs intercept	Assumed to be zero at 0°C
BACT	0.016	Activity costs slope	Approximated to attain range of 0 at 0°C to 0.5 at 32°C
SDA	0.15	Specific dynamic action—digestive costs	*Phoxinus* spp.; (He, 1986; Trudel & Boisclair, 1993)
FA	0.16	Egestion costs	Median value in Fish Bioenergetics 4.0 (Deslauriers et al., 2017)
UA	0.07	Excretion costs	Median value in Fish Bioenergetics 4.0 (Deslauriers et al., 2017)

In the individual‐level feeding experiment, where fish were housed in individual tanks at 21°C, maximum consumption rates were initially fit with a linear model with ln‐consumption rate as the response and ln‐mass as a predictor, as per typical scaling relationships reported in Fish Bioenergetics 4.0 (Deslauriers et al., 2017). The fitted scaling exponent was far outside the range typical for cyprinids, which we interpreted as larger fish failing to feed to true Cmax (corroborated by negative growth at the largest sizes). Because the scaling exponent could not be reliably estimated from these data, we instead estimated only the mass–consumption intercept (CA, the consumption rate of a 1‐g fish) from a linear model with untransformed consumption and ln‐mass; this intercept is anchored at the smaller fish where Cmax was achievable and is therefore retained as a usable parameter for the bioenergetics model. The scaling exponent CB was taken from a published bioenergetic model for the closely related cyprinid genus *Phoxinus* (He, 1986; Trudel & Boisclair, 1993), following the standard Fish Bioenergetics 4.0 approach when species‐specific scaling cannot be estimated.

#### 2.3.3 Bioenergetics modelling

Bioenergetics modelling was conducted with the structure of a Wisconsin Bioenergetics model, based on formulations in Fish Bioenergetics 4.0 (Deslauriers et al., 2017). All parameters, their definitions and sources are described in Table 1, and relevant equations are included in the Fish Bioenergetics 4.0 manual (http://fishbioenergetics.org). The parameterized bioenergetics model was used to generate predictions of each bioenergetic component, including fish growth in Joules and grams, amongst a range of fish masses from 1 to 10 g and temperatures of 1–33°C, in a scenario where fish were consuming Cmax, that is, the maximum physiological food consumption capacity, by setting consumption *p* = 1.0. Additionally, the model was used to generate predictions of an individual Silver Shiner over 1‐year, based on temperature regime measured using a water temperature logger located within the distribution of Silver Shiner (43.499°N, 79.777°W) collected in 2017 in Sixteen Mile Creek, Ontario (the geographic source of fish in this study; Appendix S1; Figure S5). A simulation was developed by assigning the mean stream temperature for each day of the year (1–365). Known Silver Shiner growth rates from this system were derived from empirical age and growth data (Burbank et al., 2021). A simulation was then run on a representative age‐1 fish (growth from 4.6 to 7.4 g) to attain the known growth rate with the supplied 365‐day temperature regime and bioenergetics parameters (Table 1) to estimate field consumption rate and temperature specific‐growth rate.

## 3 RESULTS

### 3.1 Metabolism

Silver Shiner metabolic rates showed varied patterns amongst lab and field in SMR and MMR (Figure 2). There was no evidence for statistical differences in SMR between lab and field data sources (linear model; *t*_4,105_ = −0.67, *p* = 0.50); therefore, lab and field SMR data were combined. Temperature and fish mass each had strong effects on SMR (Figure 2a; Table 2), but there was no evidence of an interaction between the two variables. Within warmer (18–24°C) temperature treatments where comparative data existed, there was no evidence for differences between SMR estimates between 24‐ and 48‐h lab trials (linear model; *t*_1,40_ = 0.13, *p* = 0.39). In field trials, MMR exhibited a curvilinear relationship with temperature, peaking at ~13°C and declining towards thermal extremes (Figure 2b). In these data, varied relationships occurred between water temperature, fish mass, chase time and MMR (Appendix S1, Figures S1, S2). In lab‐derived data, there was strong support for an increase in MMR with temperature (Figure 2b), but there was no support for effects of fish mass, despite measurement across a broad size range (1.89–8.20 g; Appendix S1, Figure S1; Table 2). The fitted values were used to estimate AS, in which there was a moderate curvilinear relationship with temperature, maintaining a relatively high AS throughout a wide temperature range, especially >15°C (Figure 3).

**FIGURE 2:**
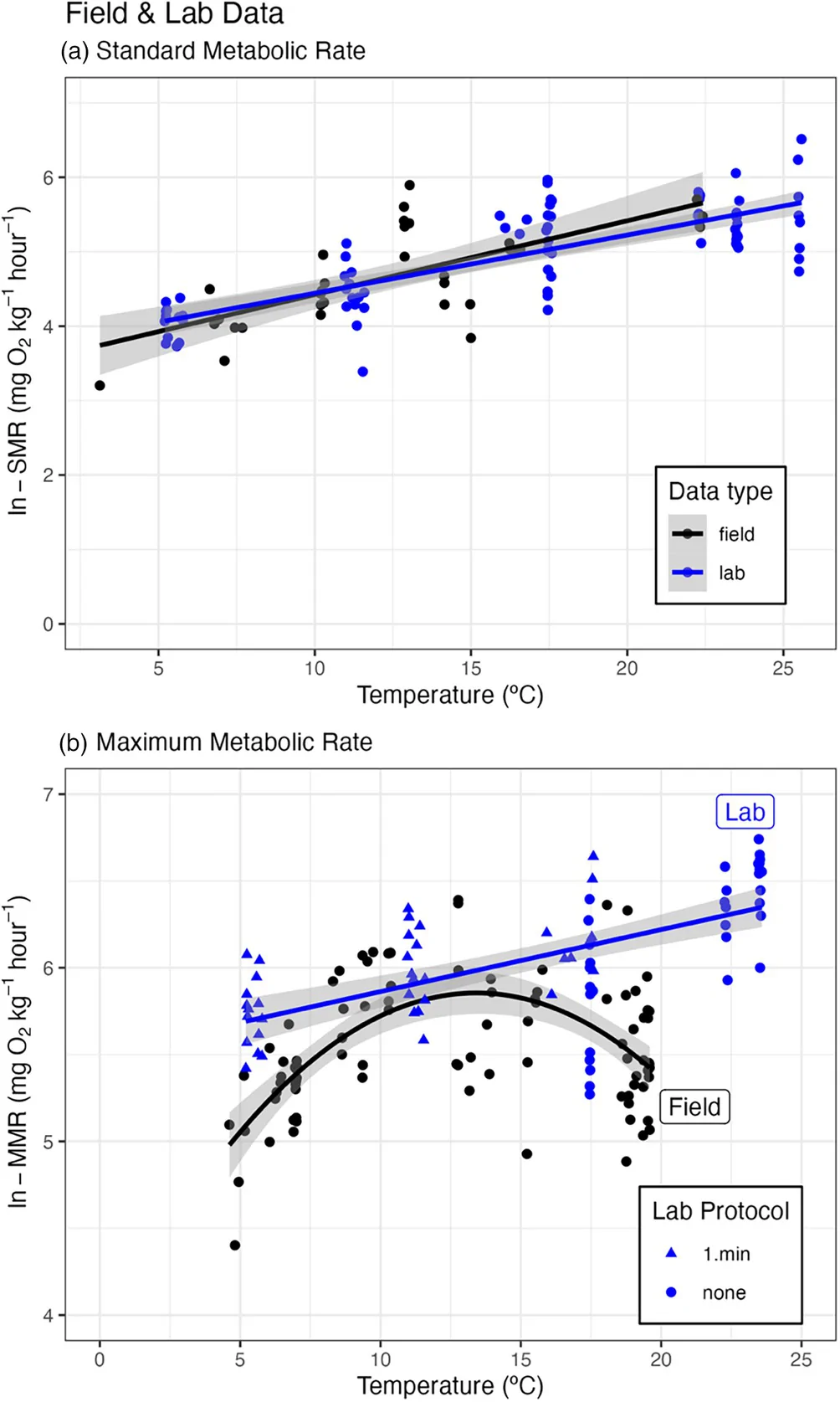
Silver Shiner metabolic rate data: (a) standard metabolic rate and (b) maximum metabolic rate measured in the laboratory and streamside in the wild. Data are fit with linear models and 95% confidence intervals. Point types refer to chase protocols (none or 1 min) in the lab; field data involved a chase‐to‐exhaustion protocol. Grey areas represent 95% confidence intervals.

**TABLE 2:** Statistical outputs from models of Silver Shiner metabolism, growth and consumption metrics.

Linear model (lab+field data): ln‐SMR ~ temperature + ln‐mass				*R* ^2^ = 0.63
Term	Estimate	SE	*t*‐value	*p*‐value
Intercept	4.10	0.17	23.6	<0.001
Temperature	0.08	0.006	13.0	<0.001
ln‐Mass	−0.284	0.09	0.09	0.001

Linear model (lab data): MMR ~ temperature + ln‐mass				*R* ^2^ = 0.56
Term	Estimate	SE	*t*‐value	*p*‐value
Intercept	201.7	69.0	2.9	0.004
Temperature	18.9	2.1	8.9	<0.001
ln‐Mass	−1.15	35.2	−0.03	0.97

Linear model: growth ~ temperature + mass + temperature×mass				*R* ^2^ = 0.57
Term	Estimate	SE	*t*‐value	*p*‐value
Intercept	−0.54	0.25	−2.18	0.03
Mass	0.14	0.04	3.70	<0.001
Temperature	0.07	0.01	4.83	<0.001
Mass:Temperature	−0.02	0.002	−6.78	<0.001

Generalized additive model: consumption ~ s(temperature)				*R* ^2^ = 0.83
Term	Estimate	SE	*F*‐value	*p*‐value
Intercept	0.08	0.004	17.73	<0.001
s(temperature)	3.94	NA	22.80	<0.001

Linear model: consumption ~ ln‐mass				*R* ^2^ = 0.80
Term	Estimate	SE	*t*‐value	*p*‐value
Intercept	0.30	0.03	9.81	<0.001
ln‐Mass	−0.12	0.02	−6.35	<0.001

**FIGURE 3:**
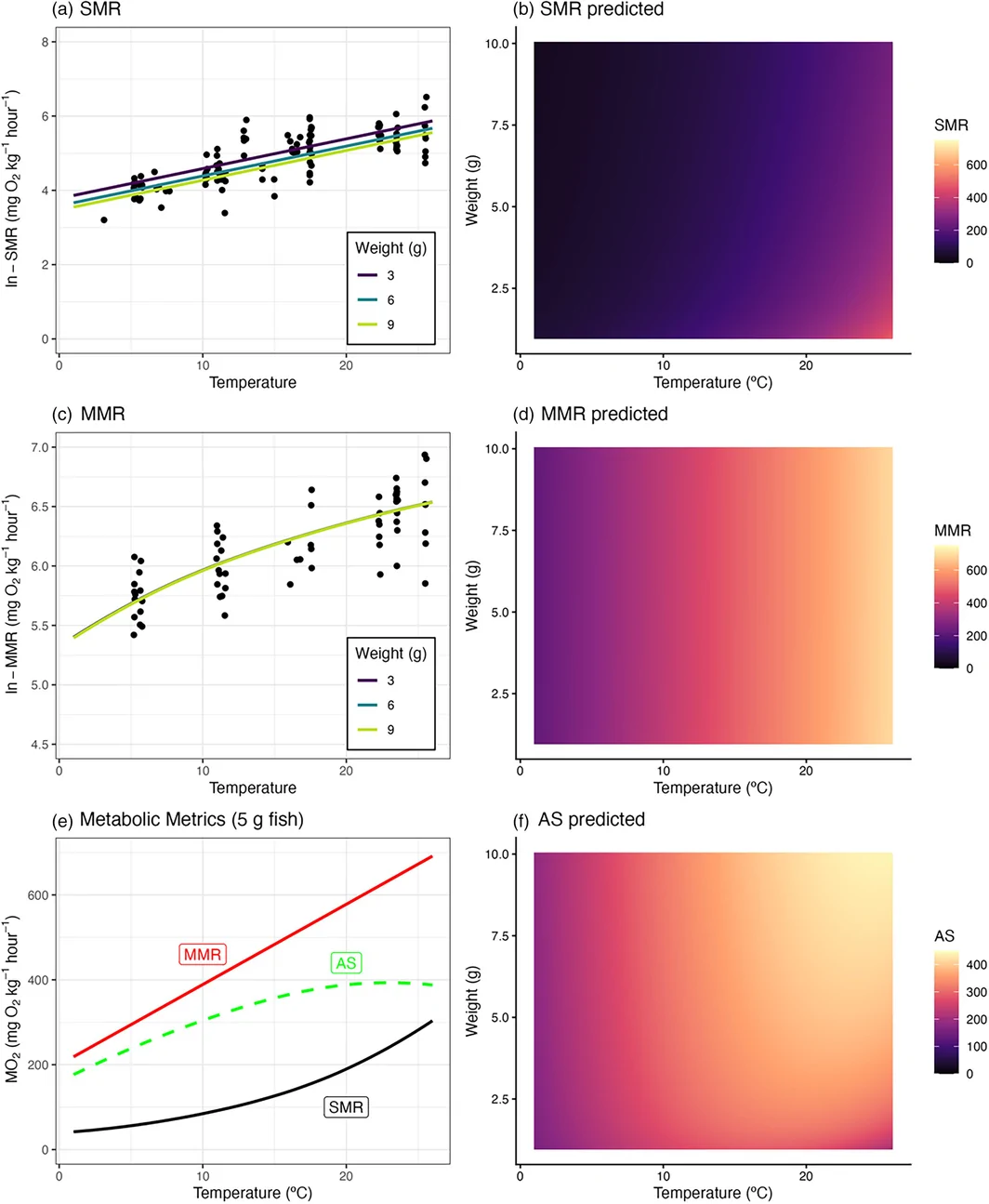
Silver Shiner metabolism metrics, (a) natural log‐transformed standard metabolic rate (SMR; dots) and predicted values (lines) by fish mass, (b) predicted SMR by temperature and fish mass, (c) natural log‐transformed maximum metabolic rate (MMR; dots) and predicted values (lines) by fish mass, (d) predicted MMR by temperature and fish mass, (e) predicted SMR, MMR and aerobic scope (AS) for a 5‐gram fish and (f) Predicted AS by temperature and fish mass. MMR data are derived from laboratory measures, SMR data are lab and field combined. Model fit metrics are reported in Table 2.

#### 3.1.1 CT_max_ & T_agitation_

During the acute thermal tolerance assessment, T_agitation_ (*n* = 6 per temperature, 23.4°C ± 2.5; mean ± SD) and CT_max_ (29.8°C ± 2.6) temperatures generally increased with increasing acclimation temperature (Figure 4). T_agitation_ was lowest at 19.8°C for fish acclimated to 6°C, increasing to the maximum observed, 25.4°C for fish acclimated to 18°C. CT_max_ was lowest at 26.2°C for fish acclimated to 6°C, increasing to its highest, 33.0°C, for 20°C acclimated fish. There was a strong effect of acclimation temperature on both T_agitation_ (linear model; *F*_3,18_ = 34.3, *p* < 0.001) and CT_max_ (*F*_3,19_ = 198.8, *p* < 0.001). Although both metrics decreased with fish size, there was no statistical evidence for an effect of fish mass on T_agitation_ (*F*_1,18_ = 1.7, *p* = 0.21) or CT_max_ (*F*_1,19_ = 0.05, *p* = 0.82). Response ratios from 6 to 20°C acclimation temperatures were 0.35 for T_agitation_ and 0.47 for CT_max_.

**FIGURE 4:**
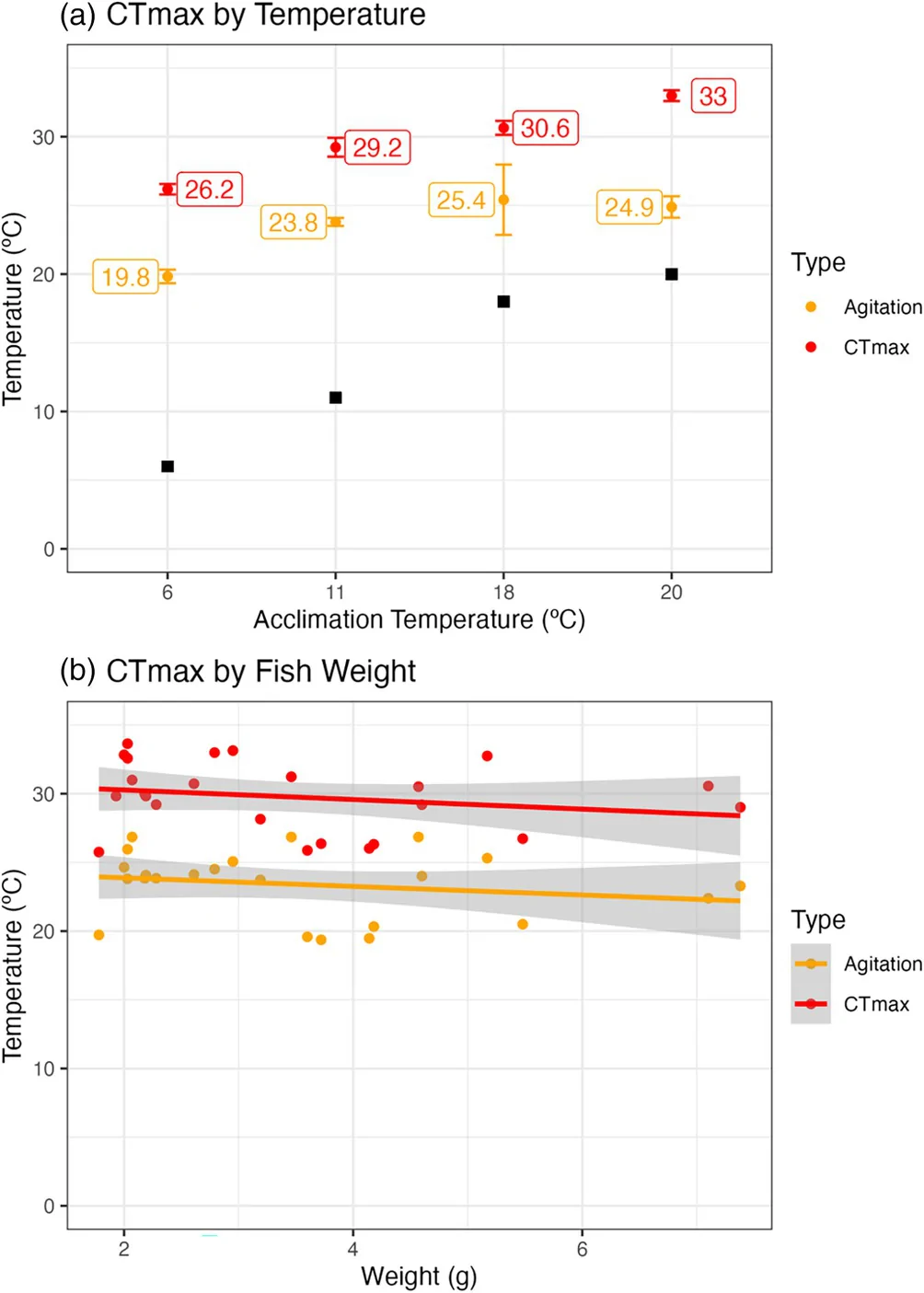
Critical thermal maximum (CT_max_) of Silver Shiner amongst (a) acclimation temperatures (black boxes). Points represent mean ± 95% confidence interval, (b) fish masses. Red points denote CT_max_ (loss of equilibrium), orange points denote agitation temperature (T_agitation_) (agitation response) and black squares denote acclimation temperature. Data are fit with a linear model ±95% confidence interval.

#### 3.1.2 Growth & food consumption

Highly variable growth patterns were observed amongst water temperatures during the 47‐day (6, 12, 18, 24°C) and 17‐day (26°C) experiment (Figure 5a). There was strong support for an interaction between water temperature and fish mass in predicting individual growth (Table 2), where smaller fish gained weight amongst all temperatures, while larger fish lost weight at an increasing rate at higher temperatures (Figure 5b). Despite being offered excess food every day, larger fish lost weight >18°C, with considerable losses up to 27% of starting weight at 26°C. Amongst all fish sizes, growth was highest at 12°C, lowest at 26°C and most variable at 24°C, where small fish gained the most weight of any temperature, but large fish lost weight (Figure 5a).

**FIGURE 5:**
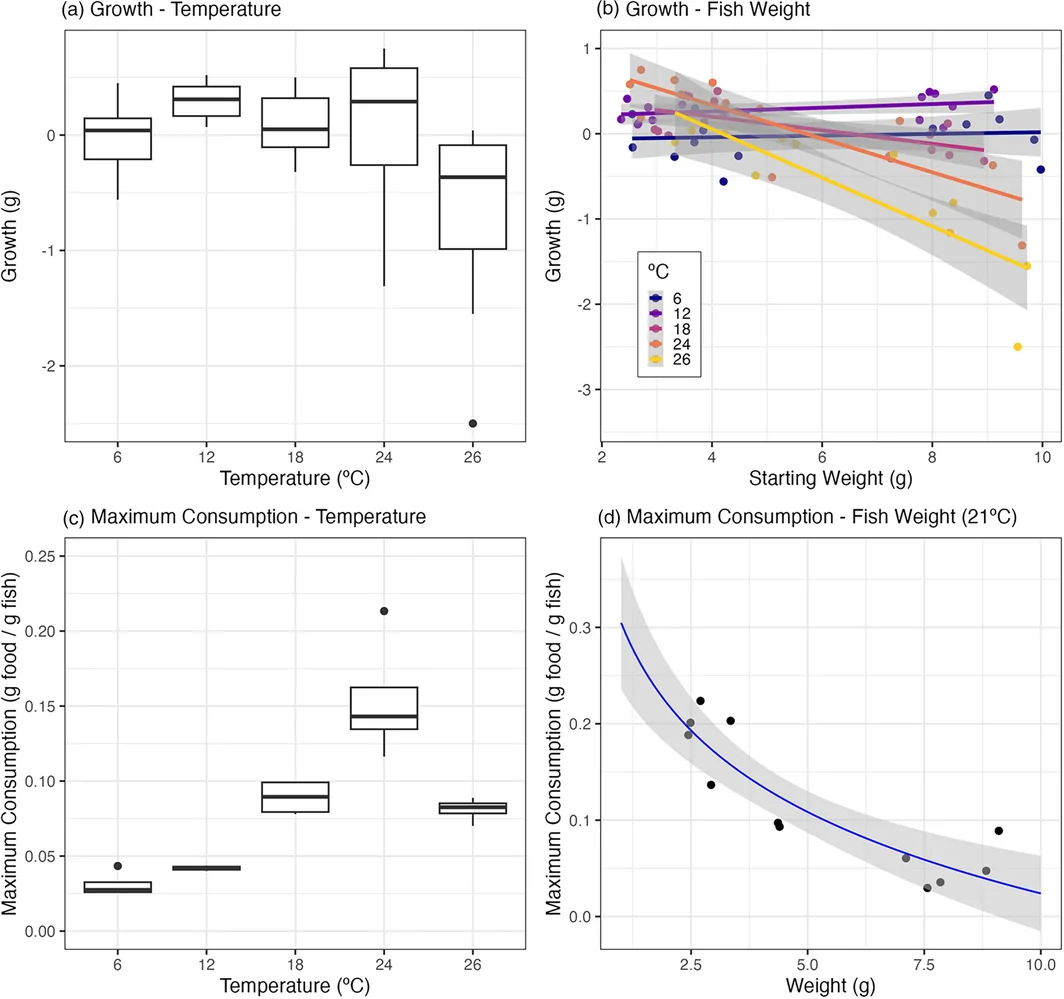
Silver Shiner feeding and growth findings: (a) Change in fish mass over time by holding temperature, (b) growth by holding temperature and fish mass, (c) maximum consumptive capacity amongst temperatures and (d) maximum consumptive capacity amongst fish sizes at 21°C. In boxplots, the central line represents the median, the box shows the interquartile range (IQR), the whiskers extend to 1.5 *IQR and the points are outliers. Fitted lines are shown ±95% Confidence Interval (CI). Model fit metrics are reported in Table 2.

Food consumption capacity increased from its lowest at 6°C (0.03 g‐food · g‐fish^−1^), peaking at 24°C (0.15 g‐food · g‐fish^−1^) and declining at 26°C (0.08 g‐food · g‐fish^−1^) (Figure 5c). Fitted with a generalized additive model, maximum consumption was estimated at 23°C (Table 2). In the individual‐level growth experiment at 21°C, consumption (per unit body weight) declined significantly with fish size, generating an intercept of 0.305 g‐food · g‐fish^−1^ for a 1‐gram fish (Figure 5d, Table 2).

#### 3.1.3 Bioenergetics modelling

A Wisconsin bioenergetics model showed a strong effect of water temperature on Silver Shiner bioenergetics (Figure 6). Based on consumption experiment outputs, food consumption capacity increased from colder temperatures up to 23°C, declining to zero at 33°C (as parameterized by CTO and CTM; Table 1). It also increased with fish mass up to a maximum of >3600 Joules day^−1^ for a 10 gram fish at 23°C (Figure 6a). Metabolic costs increased exponentially with temperature and fish mass, due to a combination of SMR and activity costs (Figure 6b). Due to the interactive effects of costs and gains, growth potential (under maximal consumption rates) was highest at 21°C, declining to negative values ≥29°C (Figure 6f,g).

**FIGURE 6:**
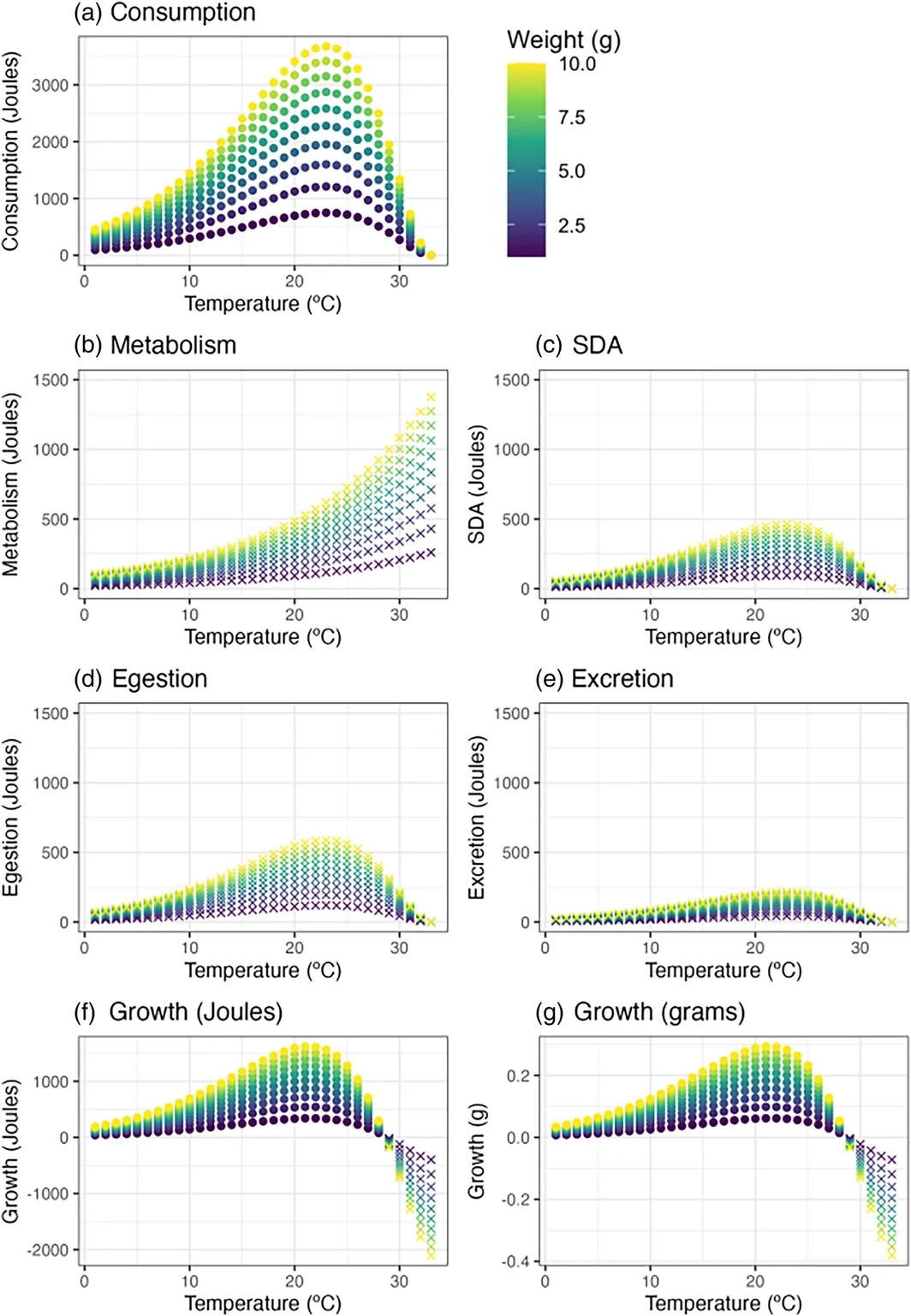
Bioenergetics model estimates for Silver Shiner (1–10 g mass), including (a) consumption, (b) metabolism costs (i.e., SMR plus activity factor), (c) digestive costs (specific dynamic action; SDA), (d) egestion, (e) excretion, (f) growth in Joules of energy and (g) growth in grams of fish mass. Circles indicate energy gains; x indicates energy losses. Model parameters are outlined in Table 1.

Applying bioenergetics models to an environmentally relevant temperature regime from Sixteen Mile Creek showed highly variable patterns of energetic costs and gains relative to water temperature throughout the year (Figure 7). In solving for the average growth rate of an age‐1 fish, the consumption p was 0.34, or 34% of maximum physiological consumption. An age‐1 fish was estimated to lose weight during the winter months when temperatures were ≤2°C and during summer when temperatures were ≥23°C, with peak field growth rates at 17°C (Figure 7).

**FIGURE 7:**
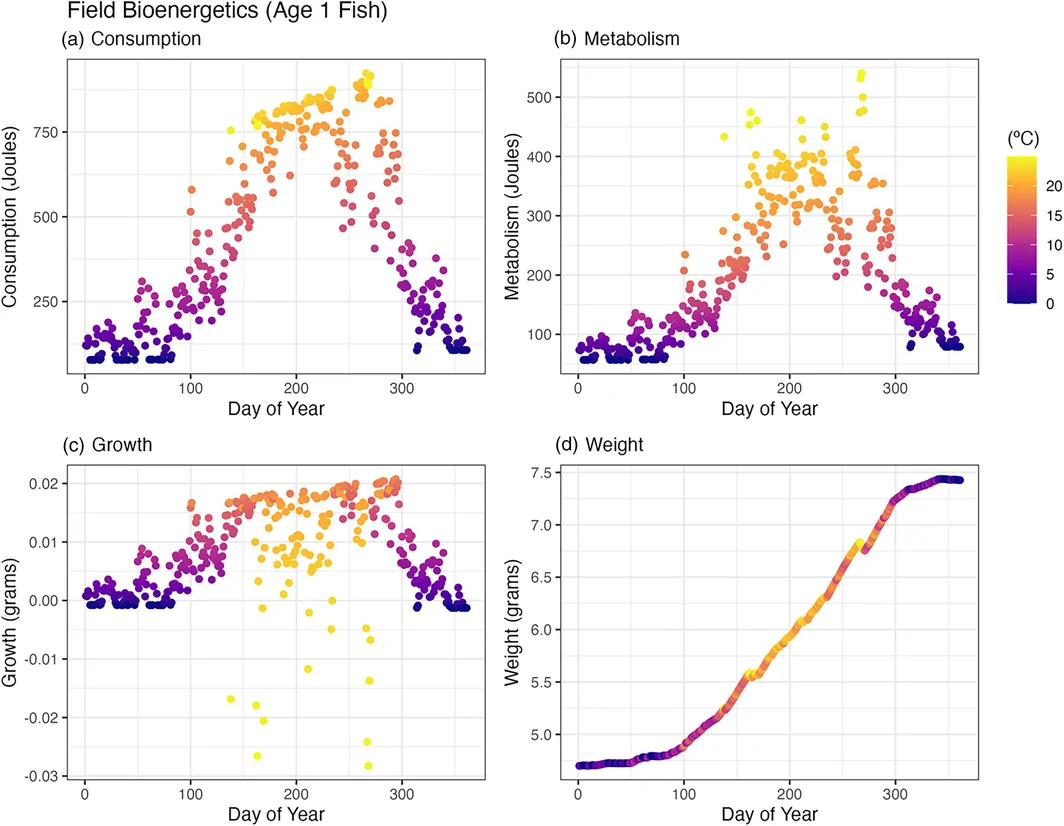
Bioenergetic model outputs for Silver Shiner applied for 1 year (ordinal date) based on daily mean stream temperatures in Sixteen Mile Creek, Ontario, including (a) consumption, (b) metabolism costs (SMR plus activity estimate), (c) fish growth and (d) fish mass over time with points coloured by temperature.

#### 3.1.4 Physiological metrics comparison

The range of physiological metrics reported above showed unique relationships with temperature, together forming a more holistic understanding of Silver Shiner thermal performance (Figure 8). Although SMR increased exponentially with temperature, MMR also increased steeply throughout an ecologically relevant temperature range, supporting relatively high AS throughout this range, peaking at 25°C (Figure 8a). Due to consistently high MMR, FAS (MMR/SMR) was highest at cold temperatures and decreased consistently with temperature (Figure 8b). Aerobic differential (AD; AS‐SMR) was positive at colder temperatures, increasing to a maximum at 14°C, with a decline to negative values ≥28°C (Figure 8c). Growth potential (at maximum consumption) increased with temperature up to a peak at 21°C, declining rapidly to negative values at ≥29°C (Figure 8d). However, estimated growth from a bioenergetics model under a realistic thermal regime and growth rate for an age‐1 fish resulted in increasing growth capacity up to 17°C, declining to negative values ≥23°C (Figure 8e). Growth in the lab followed a similar trend, but increased to a peak at 14°C, declining to negative at ≥21°C (Figure 8f). When comparing metabolic metrics (AS, FAS, AD) with growth metrics (max, field, lab), AS and FAS had varied relationships that were inconsistent with growth metrics; AD was more closely related to growth metrics (Figure 8g). In particular, peak AD was the same as peak growth in the lab (14°C), remained high at peak field growth (17°C), growth potential (21°C) and declined to zero (28°C) at similar temperature to growth potential (29°C). All physiological performance metrics were low at CT_max_ (33°C), a proxy for lethal temperature. T_agitation_ (25°C) occurred at maximum AS, but on the precipice of rapid declines in growth potential and AD with increasing temperature.

**FIGURE 8:**
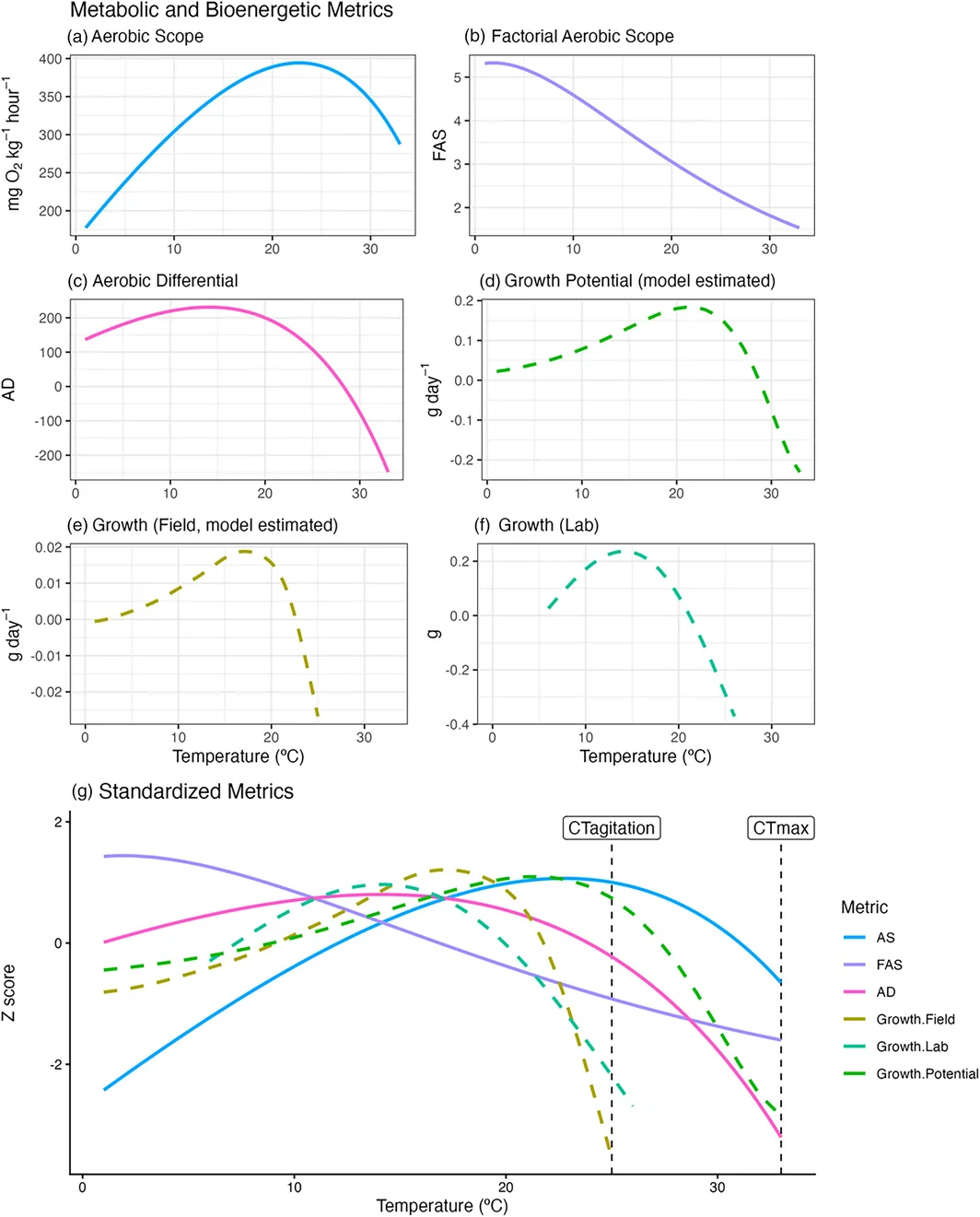
Comparison of select Silver Shiner physiological measures (predicted values derived from metabolism and growth models in Table 2, and growth measures from bioenergetics models), including: (a) aerobic scope (MMR‐SMR; AS), (b) factorial aerobic scope (MMR/SMR; FAS), (c) aerobic differential (AS‐SMR; AD), (d) growth potential (growth rate if consumption is maximized), (e) estimated field growth from a bioenergetics model, (f) growth during lab holding and (g) all measures overlaid by converting each to a standardized *z*‐score, along with T_agitation_ (temperature of agitation during the warmest CT_max_ trial), and CT_max_ (temperature of equilibrium loss).

## 4 DISCUSSION

Here we reported the field and laboratory findings from several physiological experiments on the imperilled Silver Shiner in its northern range in Ontario, Canada. Our findings showed that AD, a novel metabolic metric, was closely related to growth measures in Silver Shiner and may be of broad use when studying the growth performance of ectothermic fishes. Additionally, agitation temperatures measured from CT_max_ experiments were related to the onset of energy debt and could be an ecologically relevant indicator of thermal tolerance. Our results also showed that there is potential to collect non‐lethal, key metabolic metrics with field‐based studies, although some challenges remain with experimental design, outlined below. Lastly, we generated insights into the thermal performance of Silver Shiner, a species that can tolerate a broad temperature range, but exhibits limited growth potential and high stressor sensitivity when temperatures exceed ~21°C. Each of these components and the associated conservation implications are discussed below.

### 4.1 Physiological performance metrics

Animal physiology is complex, and there are many measures potentially relevant to a given species' eco‐physiology, performance and population success (Madliger et al., 2022; Wikelski & Cooke, 2006). Here we conducted an extensive set of experiments generating a range of physiological performance metrics related to metabolism (SMR, MMR, AS, FAS AD), acute temperature tolerance (CT_max_, T_agitation_) and bioenergetics (food consumption capacity, lab growth rate, field growth rate, maximum growth potential) and compared these metrics with the goal of gaining insights into their fundamental relatedness and relevance to the ecology and management of wild fishes, with Silver Shiner as a model species.

Much empirical research has focused on the ecological relevance of metabolic metrics, yet few studies have explored how AS or FAS directly relate to other significant endpoints such as temperature selection or growth. There is some evidence to support a direct link between AS and food consumption capacity amongst individuals (Auer et al., 2015), and studies have found that some fishes will limit their food intake to preserve a portion of AS (Jutfelt et al., 2021a, 2021b). However, other physiological processes such as digestive enzyme efficiency could also limit consumption/energy assimilation (Blier et al., 1997). Indeed, we found that Silver Shiner had a broad thermal range of AS, but consumption was more limited at both lower and upper thermal extremes, suggesting it was not limited directly by AS. Further, growth is the result of not just consumption but the net difference between gains and costs (Jobling, 1995). This was exemplified with Silver Shiner, where consumption capacity was greatest at 23°C, yet growth in the lab (observed) and field (modelled) was greatest at more moderate temperatures, 14 and 17°C, respectively.

Interpreted in the context of bioenergetics, with growth being the net result of energetic gains and costs, it is intuitive that optimal temperatures could often be more moderate than those where AS is maximized. As suggested in the energetics‐performance framework, this would be especially true under food‐limited conditions, which are thought to be common for wild animals faced with limitations due to prey availability, competition and predation (Brownscombe, Raby, et al., 2022). AS derivatives that account for these energetic trade‐offs may therefore resolve the uncertainties in AS theory. This was possibly the thrust behind FAS; however, it is not yet clear that MMR/SMR (Halsey et al., 2018) is the most appropriate transformation. With Silver Shiner, we observed no relation between FAS and any other physiological metric, including the growth metrics calculated. Here, we proposed AD (AS‐SMR) as an alternative metric, which was more closely related to growth metrics in Silver Shiner. We suggest AD is a more intuitive formulation than FAS, with the fundamental assumption that AS is a proxy for total energetic gain (e.g., from consumption capacity) and SMR a measure of costs. The potential lack of direct link between AS and consumption capacity discussed above is a likely cause of some small discrepancy between AD and growth, especially at thermal extremes. Yet, peak AD occurred at the same temperature as peak growth in the lab (estimated from models at 14°C), and modelled AD declined to zero at nearly the same temperature as growth potential (28 and 29°C respectively). Although AD was not a perfect mirror of thermal growth performance in Silver Shiner, it was more informative than AS or FAS and may be a metabolic metric with broad relevance in ecology. It could also be broadly applicable for imperilled species monitoring and recovery, if field respirometry can be effectively accomplished and fish released with high survival.

Similar to respirometry experiments to calculate AS, CT_max_ experiments are widely popular amongst physiological ecologists to estimate the upper thermal tolerance of fishes, but their ecological relevance is debated, largely due to the use of ramping rates that rarely occur in nature (Desforges et al., 2023). Physiological performance and behaviour can vary greatly prior to proxy‐lethal temperatures at CT_max_. For example, fish often show a marked increase in activity as temperatures increase during CT_max_ experiments (specifically defined by T_agitation_ and can be as much as 5–6°C lower than CT_max_), presumably due to a motivation to avoid the current temperature and seek thermal refugia (Dodsworth et al., 2025; Turko et al., 2020). Bouyoucos et al. (2023) found agitation temperature was correlated with the onset of cellular stress in Pacific Spiny Dogfish (*Squalus suckleyi*), as opposed to declines in metabolic performance. Durhack et al. (2024) found agitation temperature was also correlated with the onset of cellular stress, as well as the onset of AS decline in Brook Trout (*Salvelinus fontinalis*). We found that Silver Shiner had a mean T_agitation_ at 25°C when acclimated to 20°C, at which AS was near‐maximal, but at the onset of rapid declines in modelled growth and AD. Indeed, physiological stress has been hypothesized as closely related to energy limitation at warm temperatures (Sokolova, 2013). T_agitation_ may therefore be a useful and relatively easy to acquire metric in field and lab environments that is indicative of the onset of thermal stress and energy limitation in fishes.

### 4.2 Metabolism—Experimental design

Metabolic rate and specific measures thereof (e.g., SMR, MMR, AS) are popular focal metrics for animal physiology studies due to their relevance to animal ecology (Halsey et al., 2018; Pörtner et al., 2017). There are well‐established experimental protocols for estimating ecologically relevant fish metabolism metrics (e.g., Chabot, Koenker, & Farrell, 2016; Killen et al., 2017; Norin & Clark, 2016), yet the metabolic responses of fish in natural systems can be complex and there is value in exploring novel field‐based methods, especially for applications with imperilled species. By using a variety of lab‐ and field‐based methods, we found Silver Shiner had nuanced responses to experimental protocols that, overall, were likely reflective of higher stressor sensitivity at warmer temperatures. Small‐bodied leuciscid species, such as Silver Shiner, are generally short‐lived, rapid life history trait fishes that can have low resilience to habitat loss and other environmental stressors (Fisheries and Oceans Canada, 2022; van der Lee & Koops, 2015). We found stressor sensitivity varied between experimental protocols and was highly dependent on temperature, which posed challenges for data collection across an ecologically relevant temperature range. Specifically, commonly employed chase‐to‐exhaustion protocols in the field resulted in lower MMR estimates immediately after exercise (within 10 min) than those observed during random bouts of activity and/or stress during longer (24–48 h) static respirometry trials in the lab, especially >15°C. Indeed, this is not the only example of where spontaneous activity in respirometers results in the highest measured metabolic rates (e.g., Brieske et al., 2024). The results from field MMR trials were improbable as they suggested that Silver Shiner had no AS beyond ~21°C, yet the species maintains functional populations in a river system that routinely exceeds 25°C in summer months. Broadly, exhaustive chase protocols can underestimate true peak metabolic rate relative to swim‐tunnel and spontaneous‐activity measurements, with the magnitude of underestimation varying considerably amongst individuals (Rees et al., 2024).

Challenges with collecting MMR data in the lab were also apparent: at 18°C, the initial lab trial resulted in 4/7 individuals dying overnight in respirometers after a 1‐min chase protocol, so we adapted our methods to a no‐chase protocol at 18°C and above thereafter. These no‐chase trials resulted in significantly lower MMR estimates than 1‐min chase at 18°C; however, no‐chase respirometry at 24 and 26°C generated the highest MMR values observed in the study, demonstrating a continued increase in MMR with temperature beyond where consumptive capacity began to decline. There appears to be a thermal gradient stress response with Silver Shiner that impacts metabolic responses to respirometry protocols, and the optimal protocols for generating relevant MMR estimates. For species that are stressor‐sensitive, varied chase protocols amongst temperatures may be most effective, or even required, to acquire reasonable estimates.

For imperilled species, the capacity to bring wild animals into the lab is often limited due to conservation concerns with remaining wild populations. This was the motivation for our field metabolism trials, and it is promising that ≤5‐h streamside respirometry trials generated statistically similar SMR estimates to 48‐h lab trials, supporting the principle that non‐lethal, field‐based methods can effectively generate robust metabolism measures. Yet, these trials generated a mix of data quality; many of our streamside trials were excluded or had few data points due to low *R* ^2^ values, and neither MMR trials nor SMR trials in the field generated useful measures of MMR. Altogether, these shortcomings indicate that although field results can be comparable with laboratory results and thus provide promising applications when lab assessments of populations are not feasible, field study of imperilled species still requires cautious interpretation. In general, respirometry requires careful technical design (Killen, 2021), and challenges are exacerbated in field set‐ups, especially when managing same‐day system set‐up, experimentation and teardown that is often necessary at some sites. Admittedly, we spent considerable effort developing and refining the experimental set‐up, from initial homemade plastic respirometers that frequently broke and leaked, to professional grade chambers and modified pump impellers to generate optimal flow conditions and avoid bubble formation at cold temperatures in small chambers during trials. We believe that with the right technical expertise and experience, a range of ecologically relevant metabolism measures can be generated from non‐lethal, field‐based trials. There is certainly value in conducting field‐based trials, especially given that measures are taken from ecologically realistic conditions, and potentially with fish in a more natural physiological state. However, there are also challenges associated with this approach, in particular that capturing fish causes stress that may compound the stressors of the experimental protocols themselves, potentially biasing results. The optimal approaches and durations of these studies may be dependent on species responses and environmental context, which is worthy of further study.

### 4.3 Silver Shiner ecological energetics

Found throughout the Tennessee and Mississippi River basins of the central United States, the Silver Shiner is considered a warm‐water fish species (Fisheries and Oceans Canada, 2022). It is imperilled in Canada, with geographically disjunct populations in Ontario in riverine systems such as Sixteen Mile Creek (COSEWIC, 2011; Glass et al., 2016), the focal population for this research. Our experimental findings suggest this population is eurythermal, with a broad range of thermal tolerance and AS from near‐freezing to 33°C. While Silver Shiner appears to have the capacity to grow under a wide range of thermal conditions, warmer temperatures necessitate very high consumptive needs and are likely associated with energetic constraint and higher sensitivity to stress. Acute thermal tolerance findings suggest that Silver Shiner has a CT_max_ and temperature response ratios slightly below median values for freshwater fishes, which have been proposed as indicators of climate change sensitivity (Comte & Olden, 2017; Morley et al., 2019).

There has been some uncertainty about Silver Shiner maximum age, with studies estimating 4 years and 12 years (Burbank et al., 2021; Young & Koops, 2013). Bioenergetics modelling suggests that the formation of false growth annuli in the summer is possible, especially with warmer temperatures and limited food, which could result in periods of neutral or negative growth, and therefore overestimate maximum age. Silver Shiner is therefore most likely short‐lived (max age ~4; Burbank et al., 2021), and age‐0 summer survival may be commonly very low (Bzonek et al., 2024). The largest fish in the consumption/growth experiments (9.8 g, 124 mm TL) were within the upper portion of the size range reported for this Sixteen Mile Creek population (max 135 mm TL; Burbank et al., 2021), meaning some fish may have been close to senescence. Available temperature data from Sixteen Mile Creek in 2017 showed summer temperatures commonly ranged from 20 to 25°C, with a maximum of 29°C recorded (Appendix S1; Figure S5). With forecasted climate change, summer stream temperatures may become a major limiting factor for Silver Shiner in this system despite exhibiting a eurythermal metabolism and existing at the northern edge of its geographic range. Our findings suggest that Silver Shiner has the metabolic capacity and overall thermal tolerance to survive up to ~32°C in the summer, but temperatures in the range of 12–21°C are more optimal for its growth, above which consumption demands and stressor sensitivity are much higher. To fuel energetic needs, this species is known to rely heavily on terrestrial invertebrates for food (Burbank et al., 2022). Although there are limited opportunities for regional environmental managers to mitigate climate change broadly, the ecology and physiology of Silver Shiner suggest that maintenance of a healthy vegetated riparian zone may be essential for its conservation by maintaining sufficient food to grow during summer and providing overhead stream cover through shading to reduce summer water temperatures. The insights gained here about the thermal ecology of Silver Shiner through several physiological experiments provide improved perspective about the role of climate change as a potential threat, as well as the characterization of critical growth habitat for the species (Fisheries and Oceans Canada, 2022).

## AUTHOR CONTRIBUTIONS

Jacob W. Brownscombe contributed to funding, conceptualization, study design, data collection, data analysis and manuscript preparation. Paul A. Bzonek and Kurtis Smith contributed to study design, data collection, data analysis and manuscript preparation. Lauren E. Rowsey contributed to data interpretation and manuscript preparation. D. Andrew R. Drake contributed to funding, conceptualization, study design, data collection and manuscript preparation.

## FUNDING INFORMATION

This work was supported by Fisheries and Oceans Canada through the Freshwater Habitat Initiative Fund and Competitive Science Research Fund.

## CONFLICT OF INTEREST STATEMENT

The authors declare no conflicts of interest.

## Supporting information


**Appendix S1.** Ecological energetics of an imperilled fish species: physiological metrics, experimental protocols and implications for population recovery.
**Table S1.** Number of respirometry trials with high data quality (consistent high O_2_ *R* ^2^ values >0.9) collected from Silver Shiner in the laboratory.
**Figure S1.** Silver Shiner chase time amongst A) temperature and fish weight and B) weight and temperature, and maximum metabolic rate (MMR) amongst C) chase time and fish weight and D) chase time and temperature. Linear modelling indicated water temperature had a strong third‐order polynomial effect on chase time (*t*_86_ = 3.25, *p* = 0.002); there was no evidence of an effect of fish weight (*t*_86_ = 0.77, *p* = 0.44). There was moderate evidence of an interaction between temperature and chase time on MMR (*t*_83_ = 2.16, *p* = 0.03), and weak evidence of an effect of fish weight (*t*_86_ = 1.70, *p* = 0.09).
**Figure S2.** Field‐based respirometry estimates of SS maximum metabolic rate (MMR) amongst temperatures and fish weights. Data are fit with a loess smooth (blue line ±95% CI).
**Figure S3.** SS metabolic rate over time from field‐based respirometry, with panels representing unique individuals and points coloured by water temperature. Prior to plotting, data were filtered to include only values with oxygen slope *R* ^2^ >0.9. Data were further filtered to only include trials with ≥3 samples for inclusion in the main publication (*n* = 31 individuals included).
**Figure S4.** SS metabolic rate over time from lab‐based respirometry, with panels representing unique individuals and points coloured by water temperature. Prior to plotting, data were filtered to include only values with oxygen slope *R* ^2^ >0.9.
**Figure S5.** Hourly water temperatures measured with a biologger located near the centre of the Silver Shiner range in Sixteen Mile Creek, Ontario, in 2017. On days with missing data, values were estimated by modelling minimum and maximum daily water tempreature values with mean, minimum and maximum air temperatures.

## Data Availability

All data and R code associated with this project are archived on Zenodo (https://doi.org/10.5281/zenodo.20614131) and also available in the GitHub repository at https://github.com/jakebrownscombe/Silver_Shiner_Bioenergetics.
