# Supplementary material for: Ecological energetics of an imperilled fish: Physiological metrics, experimental protocols and implications for population recovery
Source: J Fish Biol. 2026 Sep 6;109(5):2627–43. doi: 10.1111/jfb.70610 (PMC13770697; doi:10.1111/jfb.70610)
Supplement: Supplementary file 1 [file JFB-109-2627-s001.docx]

Appendix S1: Ecological energetics of an imperilled fish species: physiological metrics, experimental protocols, and implications for population recovery

**Detailed Methods - Data collection**

***Field-Based Metabolism Experiments***

Each of the 11 sampling events between 28-07-2021 to 24-10-2023 occurred over a two-day period, where Silver Shiner (SS) were collected on day one using a bag seine (9 m long, 3.17 mm mesh) and identified on-site by staff trained in fish identification. Captured SS (1.8-14.2 g) were transferred into a flow-through holding bin secured in the creek, which consisted of a 95 L plastic tote with small perforations for water flow.

SS metabolism data were collected via intermittent flow-through respirometry (Killen, 2021; Svendsen, Bushnell, & Steffensen, 2016). The mobile respirometer systems were contained in two coolers (120 L) serving as water baths, with PVC conduit stands to mount four respirometer chambers in each. Initially, respirometer chambers were constructed from plastic food storage containers (15.5 x 9.0 x 6.8 cm, 725 mL) with snap-lock lids. Containers were modified with two 6.35 mm plastic PEX adapters at each end of the container sealed with non-toxic silicone for attachment of PVC tubing forming a recirculation line and a flush line. Each line was connected to a submersible water pump (Eheim 300; 5 L min^-1^; Eheim, Germany). The flush line opposite the pump was secured above the water line in the water bath to ensure no back-flushing occurred. The side of each chamber was also outfitted with a screw-tight gland, forming a port for a Firesting Optical Oxygen Probe, which was connected to two, four-channel O_2_ optical oxygen and temperature meters, which were connected to a computer running Pyro Workbench software (Pyroscience, Germany) for oxygen and temperature logging. Programmable digital timers were used to set the timing of flush pump cycles during respirometry trials. This design was updated in January 2022 to a Loligo Systems Resting Respirometry System which included: two Witrox 4-channel oxygen meters and flow-through oxygen cells (ID = 13mm) contained within the recirculation lines, and two pt100 temperature sensors placed in the two water baths. Chambers included four small (45 mm width x 85 mm length) and four medium (45 mm width x 150 mm length) cylindrical chambers, a Netio Bluetooth power strip, and the AutoResp 3 software, which enabled programmed intermittent flush and closed cycles. All initial setups were assessed for leaks by running the systems in a shallow water bath with chambers held in air, and oxygen probes were periodically calibrated prior to trials. Total volume of the chambers, recirculation lines, and recirculation pump were measured for each system setup to assign the total water volume for oxygen measurement.

On day one of each experimental period, after fish were captured and the respirometer system was established on the stream bank (~1-2 hours), field maximum metabolic rate (MMR) trials were conducted. Fish were individually dip-netted from the flow-through holding container and placed in a circular plastic pool (1 m diameter, 20 cm water depth). Fish were then chased by hand or with dip nets by two researchers to coerce constant swimming until fish no longer attempted to escape the approach. Total chase time was recorded, and each fish was immediately transferred as quickly as possible into a respirometry chamber of the appropriate size relative to fish size. The system was immediately switched from flow-through to a closed cycle to measure oxygen consumption for 10 minutes. SS were then removed and measured for total length and weight, and were released back into the site of capture following a brief observation period. The chamber was then flushed to reach 100% O_2_ saturation before beginning another trial. MMR trials were conducted throughout the year at ambient stream water temperatures, ranging from 3 to 21ºC and up to 12 individuals per day. MMR trials were attempted at 23ºC, but the first three fish experienced mortality, yielding no metabolism data, and further experimentation was terminated at or above this temperature.

During each experimental period, a subset of captured SS (n=10-12) not used in MMR trials were held overnight in the flow-through container within the creek to allow metabolic rates to decline and any food to be digested, prior to SMR trials beginning the following morning (9-11am). Fish were netted individually with as little disturbance as possible and transferred directly into respirometer chambers. The system was pre-programmed with the desired intermittent closed/flush cycle schedule and was placed on intermittent cycling once all the chambers were sealed. Closed/flush cycle durations varied by temperature in efforts to maintain >80% minimum O_2_ saturation during closed cycles: closed cycle durations ranged from 15 to 5 min and flush cycle durations ranged from 5 to 6 min, with shorter closed periods (and longer flush periods) at warmer temperatures. Fish were left undisturbed for 4-5 hours for oxygen measurement, depending on the time available after capture and setup. After each day of SMR trials, a single chamber was left empty of fish for measurement of background respiration rates. Upon trial completion, SS were removed from the chambers, measured for total length (mm) and mass (g), and then released. On each SMR measurement day, habitat data at the capture site were measured including stream depth (m), substrate (% cover by class), water velocity (m s^-1^) and temperature (°C), dissolved oxygen (mg L^-1^), conductivity (μS), and pH.

***Laboratory Introduction and Care***

To conduct metabolism, consumption, growth, and CT_max_ laboratory trials, SS (n=120) were collected from Sixteen Mile Creek on two occasions (21-02-2023 and 24-10-2023), and were transported in a 311 L insulated Bonar bin with constant aeration to the Aquatic Life Research Facility (ALRF) at the Canada Centre for Inland Waters (CCIW), Burlington, Ontario. SS were held in 16 replicate 56 L aquariums that were supplied with filtered, dechlorinated municipal water at temperatures similar to that of their capture date and location. The 16 tanks were divided into four replicates of four temperature treatments and water temperatures were altered by ~1-2ºC day^-1^ to final holding temperatures (6, 12, 18, 21°C) or experimental temperatures (see below for details) via submersible heaters or water bath chillers. Equal amounts of artificial habitat and cover (i.e., rocks, PVC conduit) were placed in each aquarium along with an air stone for constant aeration. Water flow and quality (i.e., temperature, DO%, NH_4_/NH_3_, pH) were monitored and maintained either via recirculation with a 300 L water sump for each temperature rack, or via flow-through water from the facility, depending on temperature. SS were monitored daily for condition (i.e., fungal growth, disease, other stress indicators) and were fed a diet of bloodworms or brine shrimp (Sally’s; San Francisco Bay Brand) once or twice per day, depending on staff availability. SS readily ate both food types within 1-2 days of introduction but appeared to prefer bloodworms. Feeding amounts were monitored and varied over time to ensure a moderate excess of food remained after each feeding (i.e., fed to satiation). Tanks were siphoned 30-40 minutes after daily feeding to remove waste.

***Laboratory Metabolism Experiments***

Laboratory respirometry trials followed similar methods to the field metabolism trials using the Loligo respirometry equipment, though were extended for 24-48 hours. For the first batch of fish, trials were conducted from 06-03-2023 to 06-04-2023, and the second batch of fish from 19-12-2023 to 27-02-2024. Prior to respirometry trials, fish were held in lab at acclimation temperatures for at least two weeks and fasted for a minimum of 48 hours. The same respirometer systems from field trials were used and held in a temperature-controlled environmental chamber room in ALRF. Submersible heaters were added to the water baths to stabilize temperatures and provide redundancy. The first respirometry trials included acclimation temperatures 6, 12, 18, and 23ºC, with no chase protocol used. The second respirometry trials were conducted at 6, 12, 18, 24, and 26ºC with a standardized 1-minute chase protocol used to elicit MMR (similar to field methods) prior to placing fish in the chambers. However, during the first trial at 18ºC, 4/7 fish experienced mortality within the chambers overnight, presumably caused by higher stress at warmer temperatures. Chase protocols were therefore not conducted in the subsequent 18ºC trials or at any higher temperatures due to the inability to effectively collect metabolism data post chase protocol at these temperatures. Trials were conducted mainly for 48 hours; however, due to logistical constraints, some (n=30/78) were limited to 24 hours. The effect of this was compared statistically below. Upon trial completion, fish mass and total length were recorded, and they were transferred back into their aquariums. Unfortunately, in the first batch of trials, bubble formation within the chambers was a persistent issue at colder temperatures, yielding unusable respiration data at 6 and 12ºC. This was remedied in the second batch of trials by removing the ball valves in pump lines, instead moderating flow by cutting down the tines on the pump impellers. We hypothesize that ball valves produced a pressure differential within the systems that resulted in problematic bubble formation in small respirometer chambers at cold temperatures. The total usable trials are reported in Table S1.

Table S1: Number of respirometry trials with high data quality (consistent high O_2_ R^2^ values >0.9) collected from Silver Shiner in the laboratory.

| Temp (°C) | 48-hour | 24-hour | Total |
| --- | --- | --- | --- |
| 5 | 4 | 0 | 4 |
| 6 | 10 | 0 | 10 |
| 11 | 7 | 0 | 7 |
| 12 | 7 | 0 | 7 |
| 17 | 7 | 10 | 17 |
| 18 | 0 | 5 | 5 |
| 22 | 7 | 0 | 7 |
| 24 | 6 | 7 | 13 |
| 26 | 0 | 8 | 8 |

***CT_max_ Experiment***

To investigate SS thermal tolerance, a subset (n=24) of the first batch of SS introduced to the lab were subjected to CT_max_ experiments between 02-05-2023 and 05-05-2023, CT_max_ trials were conducted in the same temperature-controlled environmental chamber room used for the laboratory metabolism experiments. Trials were conducted on six fish at a time. Fish were divided into two 56 L aquariums that were plumbed to circulate water from a 60 L sump tank. The sump tank was supplied with constant aeration to maintain high oxygen saturation levels. Before each trial, fish were fasted for 24 hours and acclimated to the experimental tanks overnight, beginning at holding condition temperatures: 6, 12, 18, or 20ºC (at the time of CT_max_ trials, the temperature of the 21°C holding tanks were closer to 20°C). During the trial, water temperature was increased at a rate of 0.3°C min^-1^, which is most commonly used to assess the acute thermal tolerance of fish (Desforges *et al.*, 2023; Raby *et al.*, 2025). The times and water temperatures for T_agitation_ and CT_max_ were recorded for each fish by a visual observer. Following literature descriptions of agitation temperature in fish, T_agitation_ was identified when SS exhibited marked erratic behavior, agitated swimming, and rapid ventilation for >5 seconds (McDonnell and Chapman, 2015). CT_max_ was determined as the temperature at which individuals lost equilibrium. A single observer monitored all 6 fish concurrently for signs of agitation or equilibrium loss.

During trials, the ramping of water temperature was controlled manually by operating 3 heaters placed inside the shared sump tank. For each acclimation temperature trial, a practice trial was first conducted without fish to optimize the volume of water in the sump and the timing of switching heaters on. Aquarium water temperature was monitored with the same Loligo Systems Resting Respirometry System described in the respirometry experiments. The rate of warming was monitored in real time with a shiny application (version 1.8.1; Chang et al., 2024) developed in R (version 4.2.1; R Core Team, 2019) to display the current water temperature, the current rate of temperature increase and the average rate of temperature increase over the duration of the trial. Across all trials we maintained a warming rate of 0.29 ± 0.1 °C min^-1^ (mean ± sd), and a slope R^2^ of 0.99 ± 0.01.

***Growth & Consumption Experiment***

A ~7 week (47 days total) growth and consumption experiment was conducted with the second batch of SS introduced in the lab, with n=15 SS per acclimation temperature treatment (6, 12, 18, 24°C) distributed amongst four tanks. After 12 days of temperature acclimation, SS initial fish lengths and weights were measured on the live fish in air using an electronic mass balance ±0.01 g on 03-11-2023. However, two individuals from the 24°C treatment died shortly after initial weighing, reducing to n=13. Beginning on day 1 of the experiment, SS were fed an excess diet of brine shrimp once daily then transitioned to brine shrimp or bloodworms fed twice per day on weekdays and once on weekends (bloodworms every morning feeding) on day 20 of the experiment. Food (bloodworms and brine shrimp) was packaged suspended in water in frozen cubes, so actual food wet weight for each food type was quantified (n=5 cubes per food type) by measuring the frozen weight, then thawing and straining the cubes through a fine-mesh filter for 5 minutes, allowing all excess water to drain. The bags were inverted, and the feed was removed using forceps and placed in beakers for weight measurement, which was 46.39% of frozen weight for bloodworms, and 55.26% for brine shrimp. All food amounts were measured frozen and converted to wet weight as a measure of food consumption relative to fish wet weights. During the growth experiment, the amount of food provided to each tank was measured each day. In addition, for a subset of days (n=7 consecutively, 05-12 to 11-12) within this period, the percentage of feed (exclusively bloodworms) consumed was measured for each tank. This was completed by siphoning all wastes out of the tank prior to feeding, measuring the food provided to each tank, then after ~45 minutes, siphoning and collecting remaining food for measurement. Remaining food was filtered in the same manner as frozen food above for measurement of total wet weight per tank, which was subtracted from the initial wet feed amount to calculate food consumed per tank. Upon experiment completion, SS were then fasted for 72 hours and final mass was measured in the same manner as initial weights on 19-12-2023.

After the initial growth and consumption experiment at 6-24ºC, the experiment was repeated with a subset of fish at 26ºC to further assess whether consumption and growth decline at the upper end of ecologically relevant temperatures for this species. Because larger fish in the 24ºC treatment had lost weight, individuals (n=12) were selected from the 12ºC and 18ºC treatments, separated into four replicate tanks and temperatures were increased 1-2ºC day^-1^ to 26ºC on 05-02-2024. The 26ºC experiment was performed in the same manner as above from 08-02-2024 to 23-02-2024, with wastes and feed (exclusively bloodworms) consumed measured from 19-02-2024 to 23-02-2024. On the same timeline as the 26ºC experiment, the remaining SS from 12ºC were separated into individual tanks (n=1 fish per tank, n=12 tanks) for an individual-level growth experiment to assess the effects of fish size on consumptive capacity at 21ºC. This temperature was chosen to attain near-peak consumptive capacity (based on the first temperature-consumption experiment). The feeding, waste weighing, and fish weighing timeline was the same as the 26ºC treatment. Upon the completion of 21ºC and 26ºC experiments, all SS were fasted for 48 hours, euthanized with 250 mg L^-1^ tricaine methanesulfonate, and a final weight was measured.

**Data Analysis**

***Metabolism Experiments***

Unprocessed respirometry data were exported from the program software (Firesting or Loligo), and all subsequent analyses were conducted in R. Oxygen values (mg L^-1^) from each closed period were filtered to exclude a 1-min buffer at the start and end of each period, and remaining data were fit with linear models to derive slopes and R^2^ values for fish and background oxygen consumption. Slopes were converted to mg O_2_ by multiplying by total respirometer volume, including the chamber, recirculation lines and pump, minus fish volume. With all respiration data, the data were filtered to include slopes with R^2^>0.9, and fish:respirometer volume ratios of <120:1. For field metabolism trials, background consumption estimates for the corresponding day were subtracted from fish data to generate corrected fish O_2_ consumption values. In the lab, variance was observed amongst respirometers and each respirometer was not measured each day, so we elected to apply a temperature-level mean background value to each trial. Corrected O_2_ values were divided by fish mass; all metabolism data are expressed in mg O_2_ kg^-1^ hr^-1^ (MO_2_). MMR was estimated from the value of the 10-minute trial (field MMR) or the highest value from entire 24-48 hour trial (lab). SMR was calculated from lab data by removing the five lowest MO_2_ values then calculating the mean of the lowest 10% of MO_2_ values (Chabot *et al.*, 2016). Field SMR data were more limited in sample size, and no obvious low outliers existed, so the mean of the three lowest MO_2_ values was taken as an estimate of SMR.

There were clear differences between the relationships between temperature and MMR between field and lab trials, so lab and field MMR were modeled separately. With field-based MMR data, linear models were fit to assess relationships between chase time, fish mass, temperature, and MMR. SS chase time was fit with temperature (third order polynomial) and fish mass as predictors. MMR was ln-transformed and fit with chase time, temperature (second order polynomial), and ln-mass. Lab MMR data were fit with temperature and ln-fish mass predictors. As described above, substantial mortalities were observed in the lab trials from a 1-min chase at 18ºC, so chase protocols were terminated thereafter. Therefore, MMR estimates from 18ºC were compared between chase and no-chase data with an independent t-test. Due to significantly lower MMR values in no-chase data, only chase data were included in the final lab MMR model at 18ºC. To further assess the data from higher temperatures, a linear model was fit to MMR with data type (chase only data, chase only data plus >20ºC no chase data), temperature, and their interaction as predictors. There was no support for the effect of data type or interaction, indicating including higher temperature values had limited influence on the derived MMR estimates, and no-chase data from >20ºC were therefore retained. To compare field and lab SMR data (Figs. S3, S4), ln-SMR was fit with temperature, ln-mass, and data type (field, lab) as predictors. A second linear model was also fit with metabolic rate data converted to g-O_2_ g-fish^-1^ day^-1^ to generate estimates at the appropriate scale for bioenergetic models below. Some lab trials were limited to 24 hours instead of 48 hours; this potential effect was assessed by filtering the lab data to include temperatures that contained both durations (16-24ºC) and fitting a linear model to MO_2_ with measure duration as a categorical predictor.

***Bioenergetics Modeling***

Bioenergetics modeling for SS was conducted with the structure of a Wisconsin Bioenergetics model, based on formulations in Fish Bioenergetics 4.0 (Deslauriers *et al.*, 2017). This model follows the formula: Consumption = Metabolism (SMR + Activity + SDA) + Wastes (Egestion + Excretion) + Growth, where SMR is standard metabolic rate, Activity is active metabolism costs, and SDA is specific dynamic action (digestive costs). Parameters were derived from the above metabolism and consumption experiments, supplemented with values from the literature where needed. All parameters, their definitions and sources are described in Table 1, and relevant equations are included in the Fish Bioenergetics 4.0 manual (http://fishbioenergetics.org). Respiration equation 1 was used, producing an exponential increase in metabolic costs through an ecologically-relevant temperature range. Consumption equation 2 was used, generating a curvilinear relationship between consumption and temperature, consistent with the relationship observed during the feeding experiment. Wastes equation 1 was used, generating consistent proportions of waste products per consumption rate amongst temperatures and fish masses.

The parameterized bioenergetics model was used to generate predictions of each bioenergetic component, including fish growth in Joules and grams, amongst a range of fish masses from 1-10 g and temperatures of 1-33ºC, in a scenario where fish were consuming C_max_, i.e., the maximum physiological consumptive capacity, by setting consumption p=1.0. Additionally, the model was used to generate predictions of an individual SS over 1-year, based on temperature regime measured using a water temperature logger located near the center of the SS range (43.499ºN, 79.777ºW) in 2017 in Sixteen Mile Creek, Ontario (the source of fish in this study; Fig. S5). A simulation was developed by assigning the mean stream temperature for each day of the year (1-365). Known SS growth rates from this system were derived from empirical length-at-age equations (Burbank *et al.*, 2021). These data were converted to mass-at-age using SS data from field metabolism experiments described above, using the equation: Mass = -11.03 · Length^2.76^. A simulation was then run on a representative age-1 fish (growth from 4.6 to 7.4 grams). This growth rate was achieved by running the ‘fit.p’ algorithm from Fish Bioenergetics 4.0 to determine the consumption p-value that best fits the known growth rate with the supplied 365 day temperature regime and bioenergetics parameters (Table 1). Although much more can be done with this model, this application enabled an initial assessment of how temperature affects SS bioenergetics in ecologically realistic conditions, leaving opportunities for future modeling applications.

**Figures**

Fig S1: Silver shiner chase time amongst A) temperature and fish weight, B) weight and temperature, and maximum metabolic rate (MMR) amongst C) chase time and fish weight, and D) chase time and temperature. Linear modeling indicated water temperature had a strong third-order polynomial effect on chase time (t_86_=3.25, p=0.002); there was no evidence of an effect of fish weight (t_86_=0.77, p=0.44). There was moderate evidence of an interaction between temperature and chase time on MMR (t_83_=2.16, p=0.03), and weak evidence of an effect of fish weight (t_86_=1.70, p=0.09).

Fig S2: Field-based respirometry estimates of SS maximum metabolic rate (MMR) amongst temperatures and fish weights. Data are fit with a loess smooth (blue line ± 95% CI).

Fig S3: SS metabolic rate over time from field based respirometry, with panels representing unique individuals and points coloured by water temperature. Prior to plotting data were filtered to include only values with oxygen slope R^2^>0.9. Data were further filtered to only include trials with ≥3 samples for inclusion in the main publication (n=31 individuals included).

Fig. S4: SS metabolic rate over time from lab-based respirometry, with panels representing unique individuals and points coloured by water temperature. Prior to plotting data were filtered to include only values with oxygen slope R^2^>0.9.

Fig. S5: Hourly water temperatures measured with a biologger located near the center of the silver shiner range in Sixteen Mile Creek, Ontario, in 2017. On days with missing data, values were estimated by modeling minimum and maximum daily water tempreature values with mean, minimum, and maximum air temperatures.
